# Shut Up & Write!® builds writing self-efficacy and self-regulation in early-career researchers

**DOI:** 10.1017/cts.2023.568

**Published:** 2023-05-26

**Authors:** Chelsea N. Proulx, Doris M. Rubio, Marie K. Norman, Colleen A. Mayowski

**Affiliations:** Institute for Clinical Research Education, University of Pittsburgh School of Medicine, Pittsburgh, PA, USA

**Keywords:** Self-efficacy, self-regulation, writing instruction, early-career researchers, writing productivity

## Abstract

**Introduction:**

High writing self-efficacy and self-regulation are tied to publication and grant submission. Writers with these attributes are more productive. We investigated whether participating in a Shut Up & Write!®-style intervention (SUAW) would produce statistically significant gains in writing self-efficacy and self-regulation when comparing pre-post-participation surveys.

**Methods:**

Forty-seven medical students, TL1/KL2, and early-career faculty from across the USA expressed interest in participating, with 37 completing the pre-survey. We conducted (on Zoom) a 12-week SUAW series and measured the effect using a pre-post survey adapted from the Writer Self-Perception Scale. Paired *t*-tests (α = 0.05) were conducted on three subscales to test for significant differences between pre- and post-test means. The subscales reflected writing attitudes, writing strategies, and avoiding writing distractions. Subscales showed acceptable internal consistency with Cronbach’s alphas of 0.80, 0.71, and 0.72, respectively.

**Results:**

Twenty-seven participants attended at least one session. Of these, 81% presented as female, and 60% were from NIH-defined Underrepresented Backgrounds and/or were from Minority-Serving Institutions. Twenty-four completed both the pre- and post-surveys. Sixty percent previously participated in an activity similar to SUAW. We found significant improvements in writing attitudes (*p* = 0.020) and writing strategies (*p* = 0.041) for those who previously participated. For those who had not previously participated, we found improved writing strategies (*p* = 0.002). Eighty percent were very satisfied/satisfied with SUAW.

**Discussion:**

Researchers have tied writing self-efficacy and self-regulation to timely publication and grant submission. We found significant gains in self-efficacy and self-regulation, suggesting that participation in a SUAW-style intervention may increase writing productivity.

## Introduction

Those who study the teaching and assessment of writing have found that writers with high self-efficacy – “the self-assessed ability to successfully implement writing in a specific context” (p. 1) [1] – are more productive than writers with low self-efficacy *regardless* of writing ability [2–4]. Researchers have tied writing self-efficacy to timely publication and grant submission [5]. Researchers have also consistently shown linkages between self-efficacy and self-regulation [6]. Self-regulated learners are diligent and resourceful; they tend to plan, set goals, organize, and self-monitor [6]. Self-regulation can be understood as a strategy to achieve a goal, while self-efficacy is the belief that one can successfully achieve it. It is known that these two attributes can be developed simultaneously [7].

Because submitting grant applications and publishing one’s research are essential for academic advancement across scientific fields, it is critical that those who teach and mentor early-career researchers identify writing practices that are supported by evidence [8] and encourage participation in activities that increase writing self-efficacy and its companion, self-regulation [1,9].

How writing groups operate in academia is not well understood [10], but, because they are widely believed to be beneficial in higher education [3,8,11–13], it is important to explore how they can be effectively incorporated into the training of early-career researchers. In a higher-education context, intensive writing interventions, such as summer dissertation-writing fellowships, writing retreats, and boot camps, are considered sound practices to support graduate students’ writing [14] and have been shown to produce gains in self-efficacy and self-regulation [3,14–16]. A randomized controlled trial focusing on doctoral students reported dramatic, positive changes in students’ self-efficacy and self-regulation after a 5-week writing workshop that included instruction, intensive practice in giving and receiving feedback on drafts, and writing time [8]. A study focusing on graduate student writers found small but meaningful improvements in self-efficacy and self-regulation behaviors after students participated in an intensive, week-long camp [3]. Gardner and colleagues have reported positive results gained through an intensive, years-long, structured writing intervention for biomedical graduate students that included hiring a writing specialist who taught writing seminars, facilitated writing and publishing workshops, and mentored students one-on-one [11]. These studies demonstrate that writing camps and other intensive, highly structured interventions can benefit graduate and doctoral students across disciplines [3,16], although the long-term benefits are not established [12].

Some lower-intensity interventions – which provide writing time but usually do not include instruction or peer review – have also been tried. “WAG Your Work” at Johns Hopkins describes itself as a writing bootcamp for faculty members in academia. This writing accountability group (“WAG”) activity begins with updates and goal setting, followed by 30 minutes of communal writing, then concludes with 15 minutes of reporting and wrap-up [17]. Skarupski notes increases in self-regulation behaviors following participation [18]. Vanderbilt University’s Clinical and Translational Career Development Office runs a group that writes for 25 minutes and then takes a 5-minute break before beginning again, for three cycles [19]. To our knowledge, there has been no attempt to quantify outcomes or the effect of this program on the self-efficacy and self-regulation of Vanderbilt’s participating scholars.

Whether the benefits of these writing interventions are generalizable to biomedical trainees, scholars, and early-career faculty is unknown; our literature search revealed that writing self-efficacy and self-regulation in these populations is not well studied. This may be due in part to barriers to participation. Low self-efficacy and low self-regulation themselves have been proposed as barriers to participation in writing interventions [11,16,20]. Further, we speculate that the required time commitment for many intensive interventions (days or weeks in length, or several hours daily) [3,16] might be a barrier for early-career researchers who must step away from teaching, mentoring, research, service, administrative responsibilities, and personal lives in order to participate [21,22].

With this in mind, we set out to investigate whether a relatively unstructured, low-time-commitment writing intervention (focused on mutual support but without the writing instruction, outside work, or peer review typical of high-intensity interventions) could lead to increases in writing self-efficacy and self-regulation in early-career biomedical researchers. We decided to model our less-intensive writing activity after Shut Up & Write!® [23]. Founded in San Francisco in 2007, Shut Up & Write!® (hereafter SUAW) offers in-person and online writing “meetups.” Owned and operated by a 501(c)(3) nonprofit, there is no fee to participate, and writers of any type are welcome. As of August 2022, SUAW boasted a community of 91,964 writers in 374 cities around the world [23]. Although intended for all writers, it is gaining popularity in academia [10,24]. Mewburn, Osborne, & Caldwell have reported that increasing numbers of academics are participating in SUAW groups, attracted by the “positive peer pressure, the diversity of participants, a commonality of purpose, the potential to develop networks, and the positive writing outcomes” (p. 252) [10]. Meetups can be in person or virtual.

SUAW meetups, whether in person or virtual, follow the same relatively uncomplicated structure. Unlike many other writing programs, there is no writing instruction, peer review, or critique of each other’s work. The first few minutes are devoted to a quick discussion of what each person plans to work on that day. Then, the facilitator sets a timer for an agreed-upon amount of time, usually about an hour, and each writer (when virtual) mutes themselves, shutters their webcam, and everyone writes. When the alarm sounds, everyone “returns” to the group by unmuting themselves and turning on their webcams, and the group takes turns reporting what they accomplished during the session. In some cases, participants prefer to state their goals and report their progress using the “chat” function – this can be advantageous in larger groups.

We conducted a 5-week pilot study of a SUAW-style writing intervention on Zoom during the summer of 2021. The pilot suggested that this writing intervention held promise [25]. We hypothesized that benefits of participating were cumulative and that a longer intervention would show gains in writing self-efficacy, as well as increases in self-regulation behaviors. Our main outcomes of interest were significant gains in self-efficacy and self-regulation behaviors when comparing pre-and-post-participation survey responses.

## Methods

### Study Population

We cast the net wide when recruiting participants, targeting medical students in the Gleitsman Scholars program [26], members of University of Pittsburgh TL1 and T32 programs, scholars and alumni of our Leading Emerging and Diverse Scientists to Success program [27], newly hired faculty members at the University of Pittsburgh, and TL1 and KL2 scholars from our as well as other CTSA hubs. In total, we reached out to ≈425 potential participants. Forty-seven individuals expressed interest in participating.

### Writing Activity

Our SUAW-style writing intervention began on May 23, 2022 and concluded on August 18, 2022. For the convenience of the participants, and to help ensure that we were choosing times convenient for these busy early-career researchers, days and times for meetings were chosen by the participants from Doodle Poll options. We held a total of 48, 75-minute meetings, conducted on Zoom. Participants were sent a Zoom link and passcode that was valid for every session and were encouraged to attend at least once a week, but were welcome to attend as many sessions as they chose. We followed the typical format of SUAW as described above. To close each meeting, we congratulated everyone on making and keeping this commitment to participate and encouraged them to return.

### Data Collection and Analysis

We measured the effect of this intervention on writing self-efficacy and self-regulation using a pre-post survey design. We adapted the survey Busl, Donnelly, and Capdevielle developed for their 2015 study on changes in self-efficacy and self-regulation before and after participating in graduate-student-level writing camps [3], which had itself been adapted from the validated Writer Self-Perception Scale [28]. The surveys were pre-tested during a 2021 pilot study [25] and were revised to add questions that asked about career stage, typical writing duration, number of sessions attended, and whether respondents had participated in a similar activity in the past. Our post-survey included several questions not found in the pre-survey: A 5-point Likert item rating satisfaction with the intervention, an open-ended question that asked for suggestions for improvement, a question asking participants how many sessions they attended (1–3, 4–8, 9–12, more than 12, or I did not attend), and a question asking whether they had ever attended a similar activity in the past.

We emailed a link to a survey via REDCap [29,30] (Research Electronic Data Capture) hosted at the University of Pittsburgh to those who indicated interest in participating before the first session on May 23 and then again when the study concluded on August 18. The University of Pittsburgh IRB determined this study was exempt research (STUDY22020082).

We used paired *t*-tests (α = 0.05) to test for significant differences between pre- and post-test scores. Tests were conducted on three subscales of the survey for the sample overall and after stratifying based on reported prior attendance in a similar activity. Subscale scores were calculated by taking the sum of the items for each survey section. The three resulting subscales reflected writing attitudes (self-efficacy), writing strategies (self-regulation), and avoiding writing distractions (self-regulation). Subscales showed acceptable internal consistency with Cronbach’s alphas of 0.80, 0.71, and 0.72, respectively. Participants who self-reported not attending any SUAW sessions were excluded from all *t*-tests (*n* = 4). Those who were missing a response on more than one item on a subscale were excluded.

## Results

Forty-seven trainees, scholars, medical students, and early-career faculty expressed interest in participating in the study. Thirty-seven completed the pre-survey, and 24 completed both the pre- and post-survey.

Twenty-seven individuals attended at least one SUAW session. Of these, 81% presented as female, and 60% were of NIH-defined underrepresented racial and ethnic backgrounds [31] and/or were enrolled or worked at Minority-Serving Institutions.

**Figure 1.**
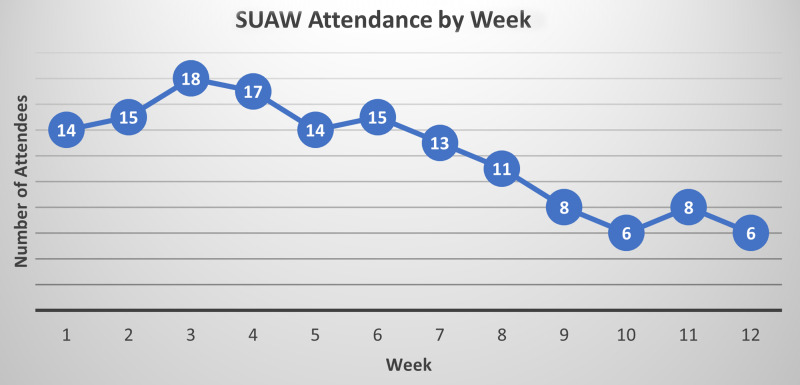
Number of unique Shut Up & Write!® attendees by week. We met for 12 weeks beginning the week of May 23 and ending the week of August 15, 2023. We did not meet during the week of July 4, 2023.

Study participants were distributed across pre-doc, post-doc, early-career faculty, and medical students, with the largest percentage being early-career faculty. Reported attendance varied, with the study’s mode attendance at 4–8 times during the 12-week study period. An average of 12 unique participants attended each week, with some attending more than one session per week. We noted attrition toward the end of the study (Fig. 1).

Four respondents reported they did not attend any of the writing sessions, although they did complete the pre- and post-survey. Reported prior attendance in a similar activity was almost evenly split among those who completed the pre- and post-survey (Table 1).

**Table 1.** Characteristics of total pre- and post-survey respondents (*n* = 24)

	Frequency (%)
**I am a/an:**	
Pre-doc	7 (29.2)
Post-doc	6 (25.0)
Early-career faculty	9 (37.5)
Medical student	2 (8.3)
**How many times did you attend this writing activity?**	
1–3	4 (16.7)
4–8	9 (37.5)
9–12	6 (25.0)
>12	1 (4.2)
Did not attend	4 (16.7)
**Before this study, have you ever participated in a similar writing activity (e.g., a "Shut Up & Write" activity, a quiet-time peer-to-peer writing group, etc.)?**	
Yes	12 (50.0)
No	11 (45.8)
I don't know	0 (0.0)
Missing	1 (4.2)

Table 2 provides descriptive statistics for pre- and post-test self-efficacy and self-regulation survey items for both individual item and *t*-test results for overall subscale scores. When asked “Please indicate your agreement with these statements on a scale of completely disagree (1) to completely agree (5),” the Writing Attitudes Subscale and Writing Strategies Subscale significantly improved from pre- to post-test. We saw no significant improvements from pre- to post-test responses for the Avoiding Writing Distractions Subscale.

**Table 2.** Descriptive statistics and *t*-test results for pre- and post-test self-efficacy and self-regulation items by subscale

	Pre-test		Post-test		*p*-value
	Mean	St. dev.	Mean	St. dev.	
**Please indicate your agreement with these statements on a scale of completely disagree (1) to completely agree (5)**					
*Writing attitudes subscale*	22.79	5.14	24.63	5.51	0.025
I understand the standard features of writing in my field.	3.90	0.55	4.05	0.69	
I am confident in my skill as a writer.	3.55	0.92	3.30	0.94	
I enjoy the process of writing.	2.85	0.93	3.00	0.92	
I consider writing to be one of my strengths.	3.05	0.97	3.32	1.00	
I have a generally positive attitude toward writing.	2.95	1.00	3.15	1.04	
When I sit down to write, I feel anxious.*	2.75	1.12	2.85	1.14	
I feel unable to manage distractions and focus on my writing.*	2.55	1.32	2.80	1.24	
I procrastinate on my writing.*	1.65	0.88	2.05	1.10	
**How often do you currently employ the following strategies in the writing process?**					
*Writing strategies subscale*	7.45	3.35	9.60	2.50	0.001
Sharing your goals	2.40	0.88	3.40	1.10	
Scheduling specific times to write each week	2.60	1.19	3.35	1.46	
Employing specific strategies to help avoid distractions (headphones, website blocking software, focus apps, etc.)	2.45	1.05	2.85	1.42	
**How often do you avoid writing or are you distracted from your writing by:**					
*Avoiding writing distractions subscale*	20.06	4.45	19.72	4.34	0.259
Social media on my computer or phone (Twitter, TikTok, Instagram, etc.)	2.90	1.17	2.55	1.43	
Research-related tasks (reading in my field, meeting with collaborators)	3.63	1.01	3.63	0.90	
Teaching-related tasks (lessons planning, grading, mentoring students)	2.55	1.28	2.60	1.35	
General work (checking work email, doing paperwork)	3.95	0.89	3.90	0.72	
My phone (emailing, texting, talking, playing games)	3.58	1.22	3.42	1.17	
Other distractions away from my computer (chatting with a friend, getting a snack, doing chores)	3.40	0.99	3.10	0.91	

* Denotes items that were reverse-coded. Items for “How often do you currently employ the following strategies in the writing process?” were rated on a scale from 1 = Never to 5 = Frequently. Items for “How often do you avoid writing or are you distracted from your writing by:” were rated on a scale from 1 = Never to 5 = Frequently.

Over half of the respondents indicated they had previously participated in an activity similar to SUAW (60%, *n* = 12) (Table 1). Subscale means for those who had previously participated showed significant improvement for both the Writing Attitudes and the Writing Strategies items from pre- to post-test. For those who had not previously participated, we found a significant improvement in Writing Strategies Subscale only (Table 3).

**Table 3.** Descriptive statistics and *t*-test results for pre- and post-test self-efficacy and self-regulation items by subscale, stratified by prior participation in a similar writing activity

	Pre-test		Post-test		*p*-value
	Mean	St. dev.	Mean	St. dev.	
**Those who have participated in a writing activity before:**					
Writing attitudes subscale (*n* = 11)	21.73	1.53	24.27	1.78	0.020
Writing strategies subscale (*n* = 12)	7.50	0.62	9.25	0.95	0.041
Avoiding writing distractions subscale (*n* = 10)	22.10	1.28	21.50	1.46	0.217
**Those who have not participated in a writing activity before:**					
Writing attitudes subscale (*n* = 8)	24.25	1.83	25.13	1.87	0.288
Writing strategies subscale (*n* = 8)	7.38	1.10	10.13	1.27	0.002
Avoiding writing distractions subscale (*n* = 8)	17.50	1.31	17.50	1.00	0.500

The question “How satisfied were you with this writing activity?” which appeared only on the post-survey (*n* = 20) had a mean of 4.15 (1 = extremely dissatisfied, 5 = extremely satisfied), with 80% of the respondents indicating they were extremely or somewhat satisfied.

The post-test concluded with an open-ended question asking for suggestions for improvement and requested comments on session length, frequency, and whether the Zoom format worked for them (Table 4).

**Table 4.** Open-ended responses to “What suggestions do you have for improving these writing sessions? Let us know if you think they should be shorter? Longer? More frequent? Did the Zoom format work for you?” (*n* = 8)

It was perfect! Great length and plenty of dates and times to choose from. The zoom format was great too, I just turned off my email notifications so it wouldn't distract me.
The sessions during the morning are more challenging due to clinical duties, I rather prefer sessions in the afternoon. The duration was adequate. The zoom format worked perfectly.
Shorter sessions that aren't in the middle of the day.
The length of time was a bit tricky when it came to scheduling. I would be helpful to be an hour (15 minutes for check and 45 minutes for writing).
Another round that would occur during the semester (as opposed to the summer when there is more travel) would be great!
The time was difficult for me to make consistently. I could only make one of the three (due to time zone differences) and even that one was difficult to protect. If the time was a little later in the morning, I could have attended more regularly. It would be nice if it was a little longer (1.5 or 2 hours). Zoom was fine. The only real problem was not having a time that works with my schedule.
I really loved these sessions – they helped me to identify a writing goal that could actually be accomplished in an hour. During these sessions, I was able to work on shorter writing tasks (as opposed to the entire Significance section in a grant). Over time, accomplishing the shorter writing tasks gave me more confidence for the longer ones.
I believe the writing session should be shorter (about 45 minutes). Once the 45 minutes mark hit I began to get distracted.

## Discussion

We investigated whether participating in a low-intensity writing intervention modeled on Shut Up & Write!® (SUAW) could produce increased writing self-efficacy and self-regulation in early-career biomedical researchers. After comparing survey responses collected before and after participation, we found significant gains in both writing self-efficacy and self-regulation. These results support findings from our 2021 pilot study [25] and strongly suggest that a low-intensity writing intervention such as SUAW can indeed produce gains in writing self-efficacy and self-regulation in early-career researchers. This is an exciting finding – not only is scientific communication a clinical and translational research core competency [32], we know that writers with high self-efficacy are more productive than writers with low self-efficacy, regardless of writing ability [2–4]. Increasing self-regulation is similarly important, as the ability to plan, set goals, organize, and self-monitor is critical in academic writing [6]. Specifically, we saw significant gains in Writing Attitudes (self-efficacy) and Writing Strategies (self-regulation), although our findings for Avoiding Writing Distractions (self-regulation) did not reach statistical significance. Why this may be so is an interesting topic for future research – it may be that it is unrealistic to expect those immersed in the “digital workplace” to unplug for any length of time [33]. Future qualitative research, perhaps interviews of participants or focus groups, may help us understand these challenges around avoiding writing distractions.

Because publishing one’s research is essential for academic advancement, those responsible for the training of early-career researchers cannot afford to neglect the cultivation of the essential attributes of self-efficacy and self-regulation [3,15,18,34]. Our study provides evidence that a low-intensity writing intervention – and not just resource and time-intensive interventions – can produce positive outcomes. These results also support our hypothesis that the benefits of participation may be cumulative, given that those who had previously participated in a similar activity showed greater gains than those who had not. Feedback on the length and timing of writing sessions, as well as the Zoom format, was quite positive, which may indicate early-career writers will continue to attend these opportunities if they are made available, and thus accumulate further benefits. Although we did not set out to measure manuscript or grant production, research findings support a relationship between writing self-efficacy, self-regulation, and writing production [2–6]. Determining the effect of SUAW on these outcomes will require longitudinal follow-up – something we plan for future research. Ideally, we would like to compare the scholarly productivity of our participants to that of a matched cohort of non-participants.

We believe this study was well-designed and conducted, although it is not without limitations. Twenty-four of 37 participants (65%) completed both the pre-and post-survey. This sample size and response rate may have resulted in a lack of power to detect some changes in self-efficacy and self-regulation. For example, we wondered if the group a participant belonged to – TL1 scholars, medical students, early-career faculty – had an effect on pre-post-differences in self-efficacy and self-regulation. Similarly, we would have liked to examine whether those who attended more sessions reported greater differences in self-efficacy or self-regulation when compared with those who attended less, but we were not powered for more sophisticated modeling. Increasing our number of participants in future SUAW sessions is key to examining these questions. We are considering modifications to our recruitment strategy. While we did adapt our survey from a validated measure, our version has not yet undergone independent validation in this population; however, the internal consistency of our subscales does support inter-relatedness among the items asked. Our surveys relied on self-reported information, and although participant responses were deidentified, the responses may nevertheless be subject to social desirability or recall bias. Additionally, others have found that females are more likely to underestimate their self-efficacy, and in our study, 81% of participants presented as female [35]. In future research, we hope to enroll enough participants to allow randomization between control and intervention groups. There may also be environmental factors at play. This study took place during a global pandemic, which may have affected participation and results in unknown ways. The study also took place during the summer months. Several regular attendees missed sessions due to vacation travel, and we observed some attrition after the US Independence Day holiday (July 4). There were one or two sessions with lower-than-expected attendance – two participants plus the facilitator. However, this did not detract from the collegial atmosphere of the meetings, and there were always enough attendees to establish a feeling of camaraderie. Finally, it would be premature to claim that the increases in self-efficacy and self-regulation we found are sustained over the long term without further study.

The defining characteristics of SUAW – the time to quietly write in the company of others, the commitment to oneself of making writing a priority by adding it to the calendar and honoring that appointment, and the act of sharing goals and accomplishments – do not require large investments of time, money, or meeting space on the part of an institution. Although the monetary investment is small, there is reason to believe that there is great value gained through providing a sense of support and camaraderie – or, as Mewburn and colleagues noted, SUAW “is really a way to create a community of practice around the process of academic writing, rather than its products” (p. 401) [24]. Similarly, Dwyer *et al*. [21] believe that these collegial writing sessions can provide an environment where early-career researchers can find pleasure in writing processes. It would be interesting to collaborate with a group of institutions to implement a virtual SUAW-style intervention for early-career researchers, which would create efficiencies for session facilitation and thus further lower any costs. A wider collaboration could potentially offer more choices of writing session length, as several participants suggested, as well as possibly alleviate time zone inconveniences. It would be interesting to run these sessions during the academic year to try to determine an optimal time for this early-career population – it may be that summer is not the ideal time for this population to participate. We will offer our next sessions during the academic year to examine whether this is a more convenient time of the year for participating early-career researchers – those who want to explore how this activity might be implemented across institutions are invited to contact the corresponding author. At the ICRE, we are actively exploring how SUAW can be incorporated into other writing activities – perhaps as a “writing lab” connected with scientific writing or grant writing courses.

Our results suggest that low-intensity writing interventions such as SUAW contribute to the development of beliefs and behaviors that will continue to serve early-career researchers as they progress in their careers [24]. Mewburn and colleagues have found “these kind of spontaneous, opportunistic writing practices can be carried on into and through the early-career researcher period and beyond” (pp. 401–402) [24]. Our findings suggest that participation in a SUAW-style intervention will promote writing self-efficacy and self-regulation for early-career researchers and that these attributes may well become ingrained and persist. Many researchers know the pain of “experience[ing] the frustration of planning to write yet never quite getting there” (p. 483) [36]. Our article contributes to the literature by reporting on a writing intervention that helps early-career researchers avoid this frustration while establishing habits of mind that will continue to serve them as they progress along their chosen career path.
